# Remotely Delivered Cardiac Rehabilitation Exercise for Coronary Heart Disease: Nonrandomized Feasibility Study

**DOI:** 10.2196/40283

**Published:** 2023-02-10

**Authors:** Oonagh M Giggins, Julie Doyle, Suzanne Smith, Grainne Vavasour, Orla Moran, Shane Gavin, Nisanth Sojan, Gordon Boyle

**Affiliations:** 1 NetwellCASALA Dundalk Institute of Technology Dundalk Ireland

**Keywords:** cardiac rehabilitation, exercise, coronary heart disease, CHD, coronary, cardiovascular, virtual rehabilitation, remote rehabilitation, digital health, heart, rehabilitation, cardiac, digital platform, digital, intervention, program, physical activity, fitness

## Abstract

**Background:**

Exercise-based cardiac rehabilitation (CR) is recommended for coronary heart disease (CHD). However, poor uptake of and poor adherence to CR exercise programs have been reported globally. Delivering CR exercise classes remotely may remove some of the barriers associated with traditional hospital- or center-based CR.

**Objective:**

We have developed a bespoke platform, Eastern Corridor Medical Engineering Centre–Cardiac Rehabilitation (ECME-CR), to support remotely delivered CR exercise. This pilot trial sought to test the ECME-CR platform and examine the efficacy and feasibility of a remote CR exercise program compared to a traditional center-based program.

**Methods:**

In all, 21 participants with CHD were recruited and assigned to either the intervention or control group. Both groups performed the same 8-week exercise program. Participants in the intervention group took part in web-based exercise classes and used the ECME-CR platform during the intervention period, whereas participants in the control group attended in-person classes. Outcomes were assessed at baseline and following the 8-week intervention period. The primary outcome measure was exercise capacity, assessed using a 6-minute walk test (6MWT). Secondary outcomes included measurement of grip strength, self-reported quality of life, heart rate, blood pressure, and body composition. A series of mixed between-within subjects ANOVA were conducted to examine the mean differences in study outcomes between and within groups. Participant adherence to the exercise program was also analyzed.

**Results:**

In all, 8 participants (male: n=5; age: mean 69.7, SD 7.2 years; height: mean 163.9, SD 5.4 cm; weight: mean 81.6, SD 14.1 kg) in the intervention group and 9 participants (male: n=9; age: mean 69.8, SD 8.2 years; height: mean 173.8, SD 5.2 cm; weight: mean 94.4, SD 18.0 kg) in the control group completed the exercise program. Although improvements in 6MWT distance were observed from baseline to follow-up in both the intervention (mean 490.1, SD 80.2 m to mean 504.5, SD 93.7 m) and control (mean 510.2, SD 48.3 m to mean 520.6, SD 49.4 m) group, no significant interaction effect (*F*_1,14_=.026; *P*=.87) nor effect for time (*F*_1,14_=2.51; *P*=.14) were observed. No significant effects emerged for any of the other secondary end points (all *P*>.0275). Adherence to the exercise program was high in both the intervention (14.25/16, 89.1%) and control (14.33/16, 89.6%) group. No adverse events or safety issues were reported in either group during the study.

**Conclusions:**

This pilot trial did not show evidence of significant positive effect for either the remotely delivered or center-based program. The 6MWT may not have been sufficiently sensitive to identify a change in this cohort of participants with stable CHD. This trial does provide evidence that remote CR exercise, supported with digital self-monitoring, is feasible and may be considered for individuals less likely to participate in traditional center-based programs.

**International Registered Report Identifier (IRRID):**

RR2-10.2196/31855

## Introduction

Coronary heart disease (CHD) is the most common cause of death globally, responsible for 16% of the world’s total deaths in 2019 [1]. Cardiac rehabilitation (CR) is a multidisciplinary intervention and is well recognized as the standard of care in CHD management. CR typically involves risk factor education, supervised exercise training, and psychological support. Numerous studies have shown that CR can aid in the recovery from an acute cardiac event and help to prevent further illness and mortality [2]. Although models vary, CR usually consists of 4 phases: phase I (in-hospital patient period; consists of education about CHD risk factors and early mobilization, with the goal of achieving functional independence at the time of discharge); phase II (postdischarge from hospital; continuing to mobilize and gradually increase functional capacity); phase III (structured exercise and education program); and phase IV (maintenance; patients receive encouragement toward maintaining an active and healthy lifestyle and continuing their exercise program). Phase III and IV CR is usually delivered in a clinical setting at hospital outpatient departments, rehabilitation clinics, or community centers. Structured exercise training is the cornerstone of both phase III and IV CR.

The benefits of exercise have been widely established in the literature, playing a key role in the primary and secondary prevention of not only CHD but a wide range of other chronic diseases such as diabetes, cancer, and depression [3-5]. CR exercise has been shown to significantly reduce all-cause and cardiac mortality compared to standard medical care without structured exercise training or advice [6]. However, despite the reported benefits, referral to and uptake of exercise-based CR are poor [7]. Multiple barriers to participation exist, such as long commutes, transportation issues, inconvenient scheduling, and work or family responsibilities [8-10]. A more recent review examined CR models based in the United States and highlighted that the reasons for low CR participation are multifactorial, with physician-, patient-, and system-related factors all being cited [11]. A suggested alternative to the traditional hospital-, clinic-, or center-based model of CR is home-based CR, where components of CR are delivered directly into the person’s home. Home-based CR increases patient accessibility and overcomes many of the obstacles that may be present with traditional center-based CR [11]. A scientific statement from the American Association of Cardiovascular and Pulmonary Rehabilitation, the American Heart Association, and the American College of Cardiology advocated for home-based CR for low-to-moderate risk patients [12], and evidence from systematic reviews comparing home- and center-based CR concluded that home-based CR programs were not inferior to center-based programs [13,14]. Using technology to deliver home-based CR has received increased interest the past decade, and this was furthered heightened with the onset of the COVID-19 pandemic.

The pandemic had a severe impact on CR services worldwide. A global survey gave an indication of the scale of the impact that COVID-19 had on CR, with approximately 75% of CR programs temporarily ceasing and other programs ceasing the initiation of new patients and reducing components delivered [15]. Many CR services changed the mode of delivery and began delivering home-based cardiac tele-rehabilitation to patients via videoconferencing platforms to curb the spread of COVID-19 infections. Cardiac tele-rehabilitation can be defined as the use of information and communication technologies, such as the internet, telephone, or videoconferencing, to deliver the components of CR completely outside of the traditional hospital, clinic, or center environment. A number of reviews have been conducted outlining the effectiveness of cardiac tele-rehabilitation intervention; however, these predominately used telephone calls for patient monitoring [16,17]. A more recent systematic review and meta-analysis have shown that home-based cardiac tele-rehabilitation is at least as effective as traditional center-based CR and, in some cases, more effective for improving exercise capacity, physical activity, quality of life, and depression scores in a population with CHD [18]. The trials included in this review largely used web-based platforms and smartphone apps delivering comprehensive home-based CR, including CHD risk factors management, physical activity, smoking cessation, medication adherence, and stress management. However, the physical activity prescription in these trials largely involved individualized physical activity programming and advice. To our knowledge, little work has been undertaken examining the remote delivery of structured CR exercise classes.

**Figure 1:**
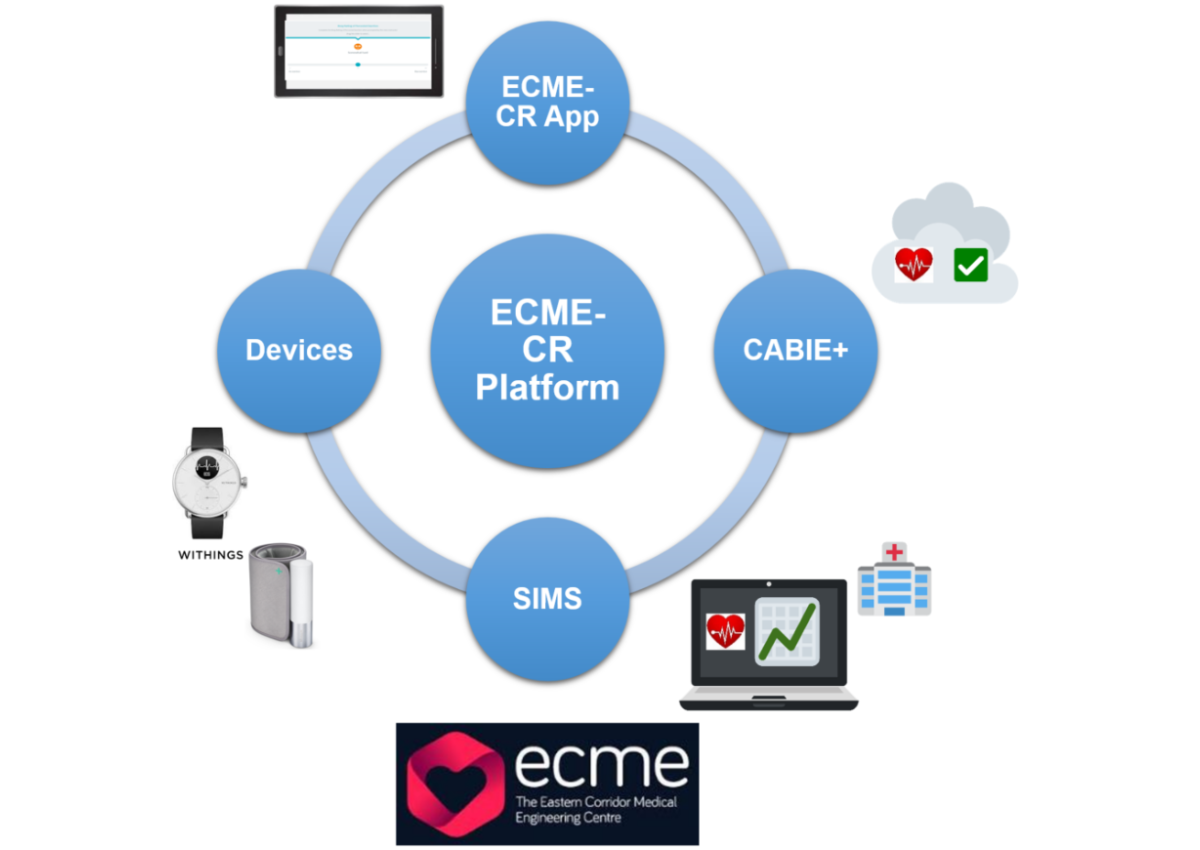
Overview of the Eastern Corridor Medical Engineering Centre–Cardiac Rehabilitation (ECME-CR) Digital Health Platform.

We have developed a bespoke, innovative solution to support the web-based delivery of CR exercise classes. The Eastern Corridor Medical Engineering Centre–Cardiac Rehabilitation (ECME-CR) digital health platform (Figure 1) has been fully described elsewhere [19]. Briefly, the platform consists of a web-based app (ECME-CR), which is used during exercise classes for guidance, monitoring, and support. Two off-the-shelf consumer devices—the Withings ScanWatch and the Withings BPM Connect—are integrated with the platform and are used to collect health and well-being data during web-based CR exercise classes as well as during the intervention period. CABIE+ is a data collection and aggregation system, which is used by the platform to organize and store the data acquired from the ECME-CR app and Withings devices. SIMS is an information management system, which is used for viewing the data collected from the app and the Withings devices in near-real time.

A pilot trial was conducted to examine the effectiveness of a remotely delivered CR exercise program supported by the ECME-CR platform in adults with CHD. We compared the effectiveness outcomes of those participating in the remotely delivered CR exercise program to a control group who participated in a traditional center-based CR exercise intervention. The protocol for this study has been described in detail elsewhere [19]. We hypothesized that the remotely delivered CR exercise program would not be inferior to the traditional center-based program.

## Methods

### Study Design

We conducted a pilot trial to examine the efficacy of a remotely delivered CR exercise program supported by the ECME-CR platform. The published protocol for this trial outlined a randomized controlled trial; however, due to the COVID-19 pandemic, a more pragmatic approach was adopted.

Participant recruitment initially commenced in August 2021; however, at this time, COVID-19 restrictions had been introduced in Ireland, which resulted in a slower-than-anticipated recruitment rate. In addition, the COVID-19 restrictions prohibited indoor group exercise classes. For this reason, those enrolled in the study at this time were not randomly allocated and participated in the remotely delivered CR exercise classes. Data collection and intervention delivery with this cohort took place between September and December 2021. In January 2022, COVID-19 restrictions had eased, and participant recruitment recommenced. This cohort of participants was randomly allocated to either the intervention or the control group, and data collection and intervention delivery took place between January and April 2022.

### Ethical Approval

The study protocol was approved by the Health and Science Ethics Committee in Dundalk Institute of Technology, and all procedures were conducted in accordance with the Declaration of Helsinki 1974 and its later amendments. All participants provided written informed consent before entering the study.

### Population and Group Allocation

The study population included participants eligible to participate in community-based phase IV CR. Participants were recruited through advertisements placed in local general practices, health clinics, and local media and through posts on social media. Potential participants made contact by telephone with the study team and were screened for their eligibility to take part by a member of the research team, over the phone, using the study eligibility criteria (Textbox 1).

**Inclusion and exclusion criteria.**

Inclusion criteria

- Men or women with documented coronary heart disease eligible to participate in a community-based cardiac rehabilitation (CR) program (phase IV CR)
- Aged 40-85 years
- Medically stable with regard to symptoms and no change in pharmacotherapy in the previous 4 weeks
- Clinical approval from their treating physician to enroll in the CR program

Exclusion criteria

- Living in a nursing home or other long-term care facility
- Have any contraindications to exercise (adapted from the American College of Sports Medicine’s Guidelines for Exercise Testing and Prescription [20]):
  Unstable angina
  Uncontrolled hypertension (ie, resting systolic blood pressure >180mmHg or resting diastolic blood pressure >110mmHg)
  Orthostatic blood pressure drop of >20 mmHg with symptoms
  Significant aortic stenosis (aortic valve area <1.0 cm2)
  Acute systemic illness or fever
  Uncontrolled atrial or ventricular arrhythmias
  Uncontrolled sinus tachycardia (heart rate >120 beats per minute)
  Acute pericarditis or myocarditis
  Uncompensated heart failure
  Third-degree (complete) atrioventricular block without pacemaker
  Recent embolism
  Acute thrombophlebitis
  Resting ST segment displacement (>2 mm)
  Uncontrolled diabetes mellitus
  Severe orthopedic conditions that would prohibit exercise
  Other metabolic conditions, such as acute thyroiditis, hypokalemia, hyperkalemia, or hypovolemia (until adequately treated)

### Cardiac Rehabilitation Exercise Program

#### Overview

Both study groups performed the same exercise program over an 8-week intervention period. Details of the exercise program are outlined elsewhere [19]. Each 60-minute session consisted of a 15-minute warm-up, 30 minutes of circuit style aerobic and strength exercises, and a 10-minute cooldown. Exercise intensity was assessed during the exercise class using the Borg scale of perceived exertion [21]. The Borg scale ranges from 6 to 20, where 6 means “no exertion at all” and 20 means “maximal exertion.” Heart rate was measured during each exercise class using the Withings ScanWatch. The ScanWatch was worn on the participants’ nondominant wrist, and for the duration of each exercise class, the ScanWatch was used in the workout mode. The intervention group undertook the exercise program in their own home, joining the CR exercise classes using Zoom videoconferencing software (Zoom Video Communications, Inc.). The control group attended a sports center in the institution to undertake their rehabilitation exercise classes.

#### Intervention Group

Participants were provided with an iPad (Apple iPad, 8th Gen, 10.2-inch, Wi-Fi, 32GB; Apple Inc.) preloaded with the ECME-CR app, the Withings devices, as well a set of free weights to use during the exercise classes. Participants received an equipment familiarization session in person at the research center, which included how to operate the iPad and the ECME-CR app, use the monitoring equipment, and record measurements. An equipment manual with written and pictorial instructions was also supplied. Participants were also provided with a mobile Wi-Fi device for the duration of the study if they did not have an established broadband internet connection in their home.

**Figure 2:**
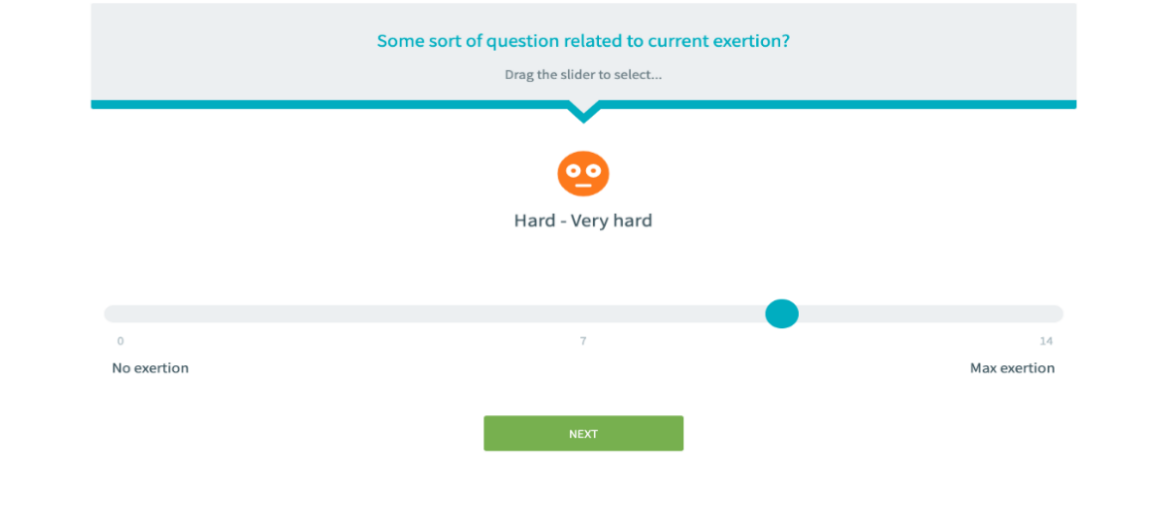
Eastern Corridor Medical Engineering Centre–Cardiac Rehabilitation (ECME-CR) app: Borg rating of perceived exertion.

Participants used the ECME-CR app during the class to record their exertion levels on the Borg scale (Figure 2). The class instructor was able to visualize and monitor in real time the exertion levels recorded by all participants simultaneously on SIMS. The instructor provided coaching and feedback on exertion when required via Zoom. Before each class, participants measured their resting heart rate and blood pressure using their BPM Connect device. This data was synchronized with the ECME-CR app and was also available for review by the instructors on SIMS before the class began, ensuring it was safe for the participant to exercise. This process was repeated at the end of the exercise class, following the cooldown period. Participants wore the Withings ScanWatch in workout mode to measure heart rate during the class. The heart rate data provided by the watch were not monitored in real time during the class. However, after each class, a summary of the heart rate data obtained was reviewed by the instructor.

#### Control Group

Each participant’s resting heart rate and blood pressure were checked using the Withings BPM Connect device before beginning each exercise class and again following the cooldown period. Participants were provided with a ScanWatch to wear for the duration of the class for continuous heart rate measurement. Self-reported exertion levels were monitored at regular intervals using the Borg scale and were manually recorded by a member of the research team.

### Outcome Measures

Outcome measures were assessed at baseline (week 0) and repeated following the intervention period (week 8). The primary outcome was cardiopulmonary exercise capacity as assessed using a 6-minute walking test (6MWT) [22]. Secondary end points were grip strength, self-reported quality of life assessed using the 12-Item Short Form Survey [23], and physical health related outcome measures, including measurement of heart rate at rest, blood pressure, and body composition. Participant adherence to the exercise program (ratio of exercise sessions completed versus prescribed) was also analyzed.

### Data Analysis

Data collected at baseline and week 8 were collated using Microsoft Office Excel (Microsoft Corp) and analyzed using SPSS software (IBM Corp). Demographic characteristics were analyzed using descriptive statistics. A series of 2 × 2 mixed ANOVAs were conducted to examine the mean differences in study outcome measures between groups (intervention group and control group) and to examine the impact of time (within subjects factor: week 0 and week 8). The Shapiro-Wilk test was applied to assess normality, and although the data were not normally distributed, ANOVA was used as it is considered to be robust to violations of nonnormality and with small sample sizes [24]. As the number of variables with missing data was low, the SPSS default for mixed within-between subjects ANOVA, listwise deletion, was used. The false discovery rate approach was used to control for type 1 error associated with making multiple comparisons [25]. Using this procedure, the *P* value was reduced by multiplying it by ([n + 1] / 2n), where n is the number of tests. This approach is recommended as it is less conservative and has greater power than the Bonferrroni correction, where *P* is divided by the number of tests [26]. A significance level of *P*<.0275 was therefore applied.

## Results

### Flow of Participants Through the Study

A total of 59 people responded to our advertisements, and 54 were contacted and screened for eligibility. In all, 28 participants satisfied the study eligibility criteria. The reason for exclusion included being unsuitable due to the use of the Withings ScanWatch in this study (ie, having a pacemaker or other implanted electronic device; n=6), uncontrolled atrial fibrillation (n=14), spontaneous coronary artery dissection (n=1), not having a CHD (n=4), or currently enrolled in a CR program (n=1). Further, 7 participants who were deemed eligible to participate were subsequently unable to enroll in the study due to family or work commitments. Of the remaining 21 participants with CHD who enrolled, 14 underwent a percutaneous coronary intervention (PCI), 3 underwent a coronary artery bypass graft, 1 underwent an aortic valve replacement and PCI, 1 underwent a PCI and coronary artery bypass graft, and 2 were treated with medication only during their hospitalization. All participants enrolled in the intervention group were urban-dwelling and had an established internet connection in their homes prior to the trial.

**Figure 3:**
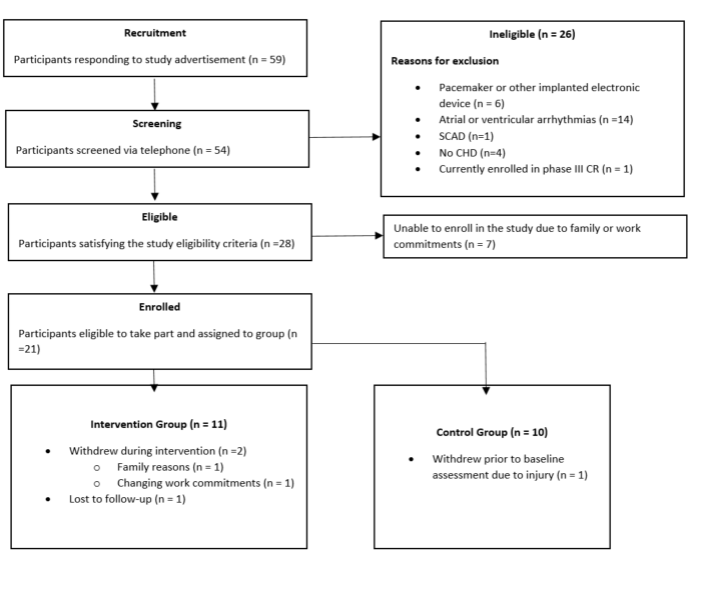
Flow of participants through the trial. CHD: coronary heart disease; SCAD: spontaneous coronary artery dissection.

In all, 11 participants were allocated to the intervention group and 10 were allocated to the control group. One participant in the intervention group was unable to attend follow-up testing due to personal reasons and was thus lost to follow-up. Two participants in the intervention group dropped out, due to family reasons and changing work commitments. One participant in the control group withdrew from the study prior to baseline measurements due to a lower limb injury. The remaining 9 participants in the control group completed the intervention. A flowchart of participants through the study is presented in Figure 3, and baseline demographics for those participants completing the intervention are presented in Table 1. Medication at baseline is also outlined in Table 1. One participant in the intervention group had a change in medication dosage (reduced dose of angiotensin-converting enzyme inhibitor) during the intervention period; all others were unchanged.

**Table 1:** Participant demographics at baseline.

Characteristic		Intervention group (n=8)	Control group (n=9)
**Sex, n (%)**			
	Male	5 (62)	9 (100)
	Female	3 (38)	0 (0)
Age (years), mean (SD)		69.7 (7.2)	69.8 (8.2)
Ethnicity, White, n (%)		8 (100)	9 (100)
Height (cm), mean (SD)		163.9 (5.4)	173.8 (5.2)
Weight (kg), mean (SD)		81.6 (14.1)	94.4 (18.0)
BMI (kg/m^2^), mean (SD)		30.4 (5.4)	31.1 (5.1)
**CHD^a^ diagnosis, n (%)**			
	PCI^b^	8 (100)	4 (44)
	CABG^c^	0 (0)	2 (22)
	AVR^d^ and PCI	0 (0)	1 (11)
	PCI and CABG	0 (0)	1 (11)
	Medication only	0 (0)	1 (11)
**Medication, n (%)**			
	Beta-blockers	6 (75)	6 (67)
	Statins	7 (88)	9 (100)
	Anti-platelets	8 (100)	8 (89)
	ACE^e^ inhibitors or ARB^f^	7 (88)	8 (89)
	Nitrates	1 (12)	1 (11)

^a^CHD: coronary heart disease.

^b^PCI: percutaneous coronary intervention.

^c^CABG: coronary artery bypass graft.

^d^AVR: aortic valve replacement.

^e^ACE: Angiotensin-converting enzyme.

^f^ARB angiotensin II receptor blockers.

### Effect of the Intervention

Table 2 shows the outcome measures assessed at baseline (week 0) and after the 8-week intervention. The primary outcome was exercise capacity as assessed using the 6MWT. One participant in the intervention group was unable to perform the 6MWT at week 8 due to a rheumatic flare-up, and therefore, these results are presented only for 7 participants in this group. The 2-way ANOVA performed revealed that there was not a statistically significant interaction effect for 6MWT distance (*F*_1,14_=.026; *P*=.87). Simple main effects analysis for the impact of time showed no statistically significant effect on 6MWT distance (*F*_1,14_=2.51; *P*=.14). No significant differences were observed following the 8-week intervention in any of the secondary outcome measurements of grip strength (right: *P*=.78; left: *P*=.29), quality of life (SF-12 physical component score: *P*=.24; SF-12 mental component score: *P*=70), resting heart rate (*P*=.89), diastolic blood pressure (*P*=.27), and body composition (weight: *P*=.17; body fat: *P*=.06; waist circumference: *P*=.55; Table 2). As no significant main effects were observed, post hoc tests were not conducted. The duration of each exercise class ranged from 50 to 60 minutes (including warm-up and cooldown), and the intensity level ranged from 6 to 14 on the Borg scale of perceived exertion.

**Table 2:** Summary of mixed between-within subject ANOVA for the intervention and control group.

	Intervention group, mean (SD)		Control group, mean (SD)			Interaction			Main effect	
	Week 0	Week 8	Week 0	Week 8	*F* test (*df*)		*P* value	*F* test (*df*)		*P* value
6MWT^a^ distance (m)	490.0 (80.2)	504.5 (93.7)	510.2 (48.3)	522.0 (49.4)	.026 (1.14)		.87	2.51 (1.14)		.14
Grip strength, right (kg)	27.9 (7.8)	26.8 (7.0)	35.3 (7.6)	35.8 (7.0)	.576 (1,15)		.46	.079 (1,15)		.78
Grip strength, left (kg)	28.5 (9.2)	28.6 (9.7)	34.6 (5.9)	36.4 (7.4)	.906 (1,15)		.36	1.323 (1,15)		.29
Weight (kg)	81.6 (14.1)	82.9 (14.2)	95.0 (17.0)	94.9 (16.7)	3.154 (1,15)		.10	2.106 (1,15)		.17
Body fat (%)	33.9 (9.8)	34.5 (9.3)	27.6 (2.4)	27.9 (2.7)	.936 (1.14)		.35	4.372 (1.14)		.06
Waist circumference (cm)	103.6 (12.8)	103.8 (12.4)	107.0 (10.1)	105.8 (12.3)	.731 (1,15)		.41	.382 (1,15)		.55
Resting heart rate (BPM^b^)	65.8 (10.1)	63.4 (8.2)	60.4 (13.8)	63.3 (10.8)	2.124 (1,15)		.17	.020 (1,15)		.89
Systolic BP^c^ (mmHg)	125.5 (11.5)	136.3 (12.0)	145.8 (21.3)	149.9 (25.7)	.293 (1,15)		.60	1.470 (1,15)		.24
Diastolic BP (mmHg)	78.1 (7.0)	78.3 (7.5)	87.9 (10.8)	82.0 (14.7)	1.436 (1,15)		.25	1.139 (1,15)		.27
SF12^d^-PCS^e^	43.8 (10.6)	41.9 (12.2)	49.7 (4.3)	46.1 (9.3)	.136 (1,15)		.72	1.500 (1,15)		.24
SF12-MCS^f^	57.9 (4.7)	56.4 (6.5)	54.1 (7.5)	57.1 (3.4)	1.381 (1,15)		.26	.153 (1,15)		.70

^a^6MWT: 6-minute walk test.

^b^BPM: beats per minute.

^c^BP: blood pressure.

^d^SF12: 12-Item Short Form Survey.

^e^PCS: physical component score

^f^MCS: mental component score.

### Program Adherence

In total, 8 (73%) of the 11 participants assigned to the intervention group completed the exercise program, attending on average 14.25 (range 12-16; adherence rate: 89.1%) out of the 16 web-based CR exercise classes delivered. In contrast, 9 (90%) of the 10 participants assigned to the control group completed the program, attending on average 14.33 (range 11-16; adherence rate: 89.6%) CR exercise classes over the 8-week intervention period. There was no significant difference in the adherence rate between the 2 groups (*P*=.84).

### Safety of the CR Exercise Intervention

There were no adverse events reported in either group participating in the CR exercise program. We did not observe any adverse cardiovascular signs or symptoms during any CR exercise class. No musculoskeletal injuries related to the intervention were reported. The need to discontinue the intervention or urgently stop exercising did not occur for any participant in this study.

### Technical Issues

Some technical issues were experienced by participants in the intervention group during the 8-week intervention period, and a log of all technical issues was maintained by the research team. The key issues encountered included difficulty logging on to Zoom for the exercise class, difficulty navigating and interacting with the features on Zoom during the exercise class (turning camera on and muting-unmuting), difficulty interacting with the Withings ScanWatch device as required during the exercise class (starting or stopping workout mode), issues synchronizing the Withings BPM Connect with the app, and difficulties interacting with the ECME-CR app as required during the exercise class. No issues with internet connection were reported by participants.

## Discussion

### Principal Findings

This pilot study sought to examine the effectiveness of a remotely delivered CR exercise program for people with CHD and compare it with a control group who participated in a traditional center-based CR exercise class. After the 8-week intervention period, neither the intervention group nor the control group showed a statistically significant improvement in 6MWT distance from baseline, which was the primary outcome measure in this study. No significant difference was found in either group in the secondary outcomes of grip strength, body composition, resting heart rate, and self-reported quality of life following the intervention period. Although weight, percentage body fat, and systolic blood pressure measurements were observed to increase from week 0 to week 8 in the intervention group, no significant effect for time emerged in this group nor in the control group.

### Comparison to Prior Work

A major trial conducted to examine the effectiveness of remotely monitored exercise-based cardiac tele-rehabilitation compared to conventional center based CR in people with CHD found that exercise capacity was comparable after a 12-week intervention period in both groups [27]. Another randomized controlled trial provided evidence of the effectiveness of a smartphone- and social media–based CR program in people with CHD [28]. Other trials have also demonstrated greater improvements in exercise capacity after home-, tele-rehabilitation–, and center-based CR [29,30]. In this study, although mean improvements in 6MWT distance of 14 m and 12 m were observed in the intervention group and the control group, respectively, at the 8-week follow-up, these improvements were not clinically significant. A 25-m improvement in the 6MWT distance has been considered clinically meaningful for patients with CHD undergoing CR [31]. This threshold was established in patients recovering from an acute cardiac event, and for patients with stable CHD, a higher threshold is likely to be required to be clinically significant. A possible explanation for the nonclinically significant improvement in this study is that the duration of the intervention or the frequency of exercise sessions may not have been sufficient to achieve an effect comparable to the results seen in other studies. Second, the age profile of participants in this trial is older (mean age: 69.5 years) than previous similar trials [18]. Previous reports have shown that improvements in exercise capacity in older adults with CHD is not as large as those observed in individuals aged <65 years [32]. Third, the inability of this study to produce significant improvements in exercise capacity compared to previous studies may indicate that the measurements used in this study were not sufficiently sensitive to identify a change in the cohort included. A previous study that examined the effect of home-based CR program in an older population with CHD also used a change in exercise capacity determined by the 6MWT as the primary outcome measure [29]. This study did demonstrate significant increases in 6MWT distance following the 3 month home-based CR program; however, the participants included had a much lower exercise capacity at baseline and therefore had a greater potential to change. Another large randomized controlled trial, which also used 6MWT distance as the primary end point, found significant improvements in 6MWT distance in participants following an 8-week smartphone-based CR program [33]. However, the trial was evaluating an early physical activity program after an acute cardiac event, where again the potential for improvement is much greater.

In this study, we observed adherence rates of >89% to the exercise program across both groups, which can be classified as high adherence [34]. Participants enrolled in this study were self-referred and were not referred by their physician, which may explain the high adherence rate observed in this study. The level of completion, that is, the number of participants with outcome data at the 8-week follow-up, was higher in the control group who were attending the center-based CR exercise class. Although a number of technical issues were experienced by participants in this group, these technical issues were not cited as a reason for withdrawal. All control group participants completed the intervention. This may be explained by the fact that COVID-19 restrictions had just been lifted when the center-based CR exercise classes were taking place. Anecdotally, participants reported that they enjoyed attending the classes as it was seen as an enjoyable social activity after a long period of social restrictions.

No serious adverse events were reported during the CR program in either group. Other investigators have found a similarly low rate of exercise-related complications during home-based CR [35]. Although some technical issues were experienced by participants in the intervention group during the exercise classes and the intervention period, these were quickly addressed and rectified by the research team. Our study, therefore, indicates that remotely delivered CR exercise at home is feasible and should still be considered for its potential for increasing overall access to CR for all eligible patients who face obstacles to traditional means of participation.

### Strengths and Limitations

This study has some limitations that should be acknowledged. First, this study was a single-center pilot trial, with a small sample size, which limits the establishment of any strong conclusions. Sample size calculations were not conducted in this pilot trial; future trials conducted will be adequately powered to determine the treatment effect. Second, few female participants participated in the study (<20%), and all participants were from the majority ethnic group in Ireland (White, Settled or non-Traveler). Although most patients attending CR and those included in other CR trials are male [36,37], the results of this trial cannot be generalized to female participants and those of other ethnicities. In this study, no participant assigned to the control group was female, and this difference, along with the differences in conditions and blood pressure between the groups, should also be acknowledged as a weakness that limits our ability to draw firm conclusions. Third, all participants included in this trial were of low-to-moderate risk, with stable CHD. The results should therefore be interpreted with caution and cannot be generalized to patients with high-risk CHD or those after an acute coronary event. Fourth, all participants in this study were self-referred and volunteered to take part. Participants referred by their physician may adhere to CR programs differently than those who self-refer, and therefore, the feasibility of our approach in a physician-referred cohort was not established. Finally, this study did not include a long-term follow-up of participants; this will be considered in future investigations. Despite these limitations, this trial provides preliminary information on remotely delivered CR exercise program supported by the ECME-CR platform.

### Conclusions

Remotely delivered CR has been suggested as an alternative to center-based CR, especially during the COVID-19 pandemic. In this trial, although both groups demonstrated improvements in the primary outcome measure of exercise capacity from baseline to 8-week follow-up, these improvements were neither clinically nor statistically significant. As previous studies have shown overwhelmingly positive outcomes for both telehealth- and center-based CR interventions, it suggests that the measurement of exercise capacity used in this study may not have been sufficiently sensitive to identify a change in the cohort of participants with stable CHD included in this study. Future work will review the exercise program delivered to both groups and use measurements that may be more sensitive to identify a change. Nonetheless, this trial provides preliminary evidence to suggest that a remotely delivered CR exercise program, supported with digital self-monitoring, is feasible and may serve as an alternative delivery model for CR for individuals less likely to participate in traditional center-based programs.

## Appendix

Multimedia Appendix 1 Data that supports the findings of this study.

## Abbreviations

6MWT: 6-minute walk test
CHD: coronary heart disease
CR: cardiac rehabilitation
ECME-CR: Eastern Corridor Medical Engineering Centre–Cardiac Rehabilitation
PCI: percutaneous coronary intervention

## Data Availability

The data that support the findings of this study are available as a supplementary file (Multimedia Appendix 1).

## References

[1] (2020). The top 10 causes of death. World Health Organization.

[2] Heran BS, Chen JM, Ebrahim S, Moxham T, Oldridge N, Rees K, Thompson DR, Taylor RS (2011). Exercise-based cardiac rehabilitation for coronary heart disease. Cochrane Database Syst Rev.

[3] Pan B, Ge L, Xun Y, Chen Y, Gao C, Han X, Zuo L, Shan H, Yang K, Ding G, Tian J (2018). Exercise training modalities in patients with type 2 diabetes mellitus: a systematic review and network meta-analysis. Int J Behav Nutr Phys Act.

[4] Vashistha V, Singh B, Kaur S, Prokop L, Kaushik D (2016). The effects of exercise on fatigue, quality of life, and psychological function for men with prostate cancer: systematic review and meta-analyses. Eur Urol Focus.

[5] Cooney GM, Dwan K, Greig CA, Lawlor DA, Rimer J, Waugh FR, McMurdo M, Mead GE (2013). Exercise for depression. Cochrane Database Syst Rev.

[6] Ekblom Ö, Cider Å, Hambraeus K, Bäck Maria, Leosdottir M, Lönn Amanda, Börjesson Mats (2022). Participation in exercise-based cardiac rehabilitation is related to reduced total mortality in both men and women: results from the SWEDEHEART registry. Eur J Prev Cardiol.

[7] Bethell H, Lewin R, Dalal H (2009). Cardiac rehabilitation in the United Kingdom. Heart.

[8] de Vos Cedric, Li X, Van Vlaenderen Ilse, Saka O, Dendale P, Eyssen M, Paulus D (2013). Participating or not in a cardiac rehabilitation programme: factors influencing a patient's decision. Eur J Prev Cardiol.

[9] Neubeck L, Freedman SB, Clark AM, Briffa T, Bauman A, Redfern J (2012). Participating in cardiac rehabilitation: a systematic review and meta-synthesis of qualitative data. Eur J Prev Cardiol.

[10] Lavie CJ, Kachur S, Milani RV (2019). Making cardiac rehabilitation more available and affordable. Heart.

[11] Chindhy S, Taub PR, Lavie CJ, Shen J (2020). Current challenges in cardiac rehabilitation: strategies to overcome social factors and attendance barriers. Expert Rev Cardiovasc Ther.

[12] Thomas RJ, Beatty AL, Beckie TM, Brewer LC, Brown TM, Forman DE, Franklin BA, Keteyian SJ, Kitzman DW, Regensteiner JG, Sanderson BK, Whooley MA (2019). Home-based cardiac rehabilitation: a scientific statement from the American Association of Cardiovascular and Pulmonary Rehabilitation, the American Heart Association, and the American College of Cardiology. Circulation.

[13] Anderson L, Oldridge N, Thompson DR, Zwisler A, Rees K, Martin N, Taylor RS (2016). Exercise-based cardiac rehabilitation for coronary heart disease: Cochrane systematic review and meta-Analysis. J Am Coll Cardiol.

[14] Taylor RS, Dalal H, Jolly K, Zawada A, Dean SG, Cowie A, Norton RJ (2015). Home-based versus centre-based cardiac rehabilitation. Cochrane Database Syst Rev.

[15] Ghisi GLDM, Xu Z, Liu X, Mola A, Gallagher R, Babu AS, Yeung C, Marzolini S, Buckley J, Oh P, Contractor A, Grace SL (2021). Impacts of the COVID-19 pandemic on cardiac rehabilitation delivery around the world. Glob Heart.

[16] Neubeck L, Redfern J, Fernandez R, Briffa T, Bauman A, Freedman SB (2009). Telehealth interventions for the secondary prevention of coronary heart disease: a systematic review. Eur J Cardiovasc Prev Rehabil.

[17] Huang K, Liu W, He D, Huang B, Xiao D, Peng Y, He Y, Hu H, Chen M, Huang D (2015). Telehealth interventions versus center-based cardiac rehabilitation of coronary artery disease: a systematic review and meta-analysis. Eur J Prev Cardiol.

[18] Ramachandran HJ, Jiang Y, Tam WWS, Yeo TJ, Wang W (2022). Effectiveness of home-based cardiac telerehabilitation as an alternative to Phase 2 cardiac rehabilitation of coronary heart disease: a systematic review and meta-analysis. Eur J Prev Cardiol.

[19] Giggins OM, Doyle J, Smith S, Moran O, Gavin S, Sojan N, Boyle G (2021). Delivering cardiac rehabilitation exercise virtually using a digital health platform (ECME-CR): protocol for a pilot trial. JMIR Res Protoc.

[20] American College of Sports Medicine (2017). ACSM’s Guidelines for Exercise Testing and Prescription. 10th ed.

[21] Borg G (1998). Borg's Perceived Exertion And Pain Scales.

[22] Bellet RN, Adams L, Morris NR (2012). The 6-minute walk test in outpatient cardiac rehabilitation: validity, reliability and responsiveness--a systematic review. Physiotherapy.

[23] Ware J, Kosinski M, Keller SD (1996). A 12-Item Short-Form Health Survey: construction of scales and preliminary tests of reliability and validity. Med Care.

[24] Sullivan LM, Weinberg J, Keaney JF Jr (2016). Common statistical pitfalls in basic science research. J Am Heart Assoc.

[25] Benjamini Y, Hochberg Y (2018). Controlling the false discovery rate: a practical and powerful approach to multiple testing. J R Stat Soc Series B Stat Methodol.

[26] Glickman ME, Rao SR, Schultz MR (2014). False discovery rate control is a recommended alternative to Bonferroni-type adjustments in health studies. J Clin Epidemiol.

[27] Maddison R, Rawstorn JC, Stewart RAH, Benatar J, Whittaker R, Rolleston A, Jiang Y, Gao L, Moodie M, Warren I, Meads A, Gant N (2019). Effects and costs of real-time cardiac telerehabilitation: randomised controlled non-inferiority trial. Heart.

[28] Dorje T, Zhao G, Tso K, Wang J, Chen Y, Tsokey L, Tan B, Scheer A, Jacques A, Li Z, Wang R, Chow CK, Ge J, Maiorana A (2019). Smartphone and social media-based cardiac rehabilitation and secondary prevention in China (SMART-CR/SP): a parallel-group, single-blind, randomised controlled trial. Lancet Digit Health.

[29] Oerkild B, Frederiksen M, Hansen JF, Simonsen L, Skovgaard LT, Prescott E (2011). Home-based cardiac rehabilitation is as effective as centre-based cardiac rehabilitation among elderly with coronary heart disease: results from a randomised clinical trial. Age Ageing.

[30] Arthur HM, Smith KM, Kodis J, McKelvie R (2002). A controlled trial of hospital versus home-based exercise in cardiac patients. Med Sci Sports Exerc.

[31] Gremeaux V, Troisgros O, Benaïm Sylvie, Hannequin A, Laurent Y, Casillas J, Benaïm Charles (2011). Determining the minimal clinically important difference for the six-minute walk test and the 200-meter fast-walk test during cardiac rehabilitation program in coronary artery disease patients after acute coronary syndrome. Arch Phys Med Rehabil.

[32] Marchionni N, Fattirolli F, Fumagalli S, Oldridge N, Del Lungo F, Morosi L, Burgisser C, Masotti G (2003). Improved exercise tolerance and quality of life with cardiac rehabilitation of older patients after myocardial infarction: results of a randomized, controlled trial. Circulation.

[33] Yudi MB, Clark DJ, Tsang D, Jelinek M, Kalten K, Joshi SB, Phan K, Ramchand J, Nasis A, Amerena J, Koshy Anoop N, Murphy Alexandra C, Arunothayaraj Sandeep, Si Si, Reid Christopher M, Farouque Omar (2021). SMARTphone-based, early cardiac REHABilitation in patients with acute coronary syndromes: a randomized controlled trial. Coron Artery Dis.

[34] Nagpal TS, Mottola MF, Barakat R, Prapavessis H (2021). Adherence is a key factor for interpreting the results of exercise interventions. Physiotherapy.

[35] Ades PA, Pashkow FJ, Fletcher G, Pina IL, Zohman LR, Nestor JR (2000). A controlled trial of cardiac rehabilitation in the home setting using electrocardiographic and voice transtelephonic monitoring. Am Heart J.

[36] Bravo-Escobar R, González-Represas Alicia, Gómez-González Adela María, Montiel-Trujillo A, Aguilar-Jimenez R, Carrasco-Ruíz Rosa, Salinas-Sánchez Pablo (2017). Effectiveness and safety of a home-based cardiac rehabilitation programme of mixed surveillance in patients with ischemic heart disease at moderate cardiovascular risk: a randomised, controlled clinical trial. BMC Cardiovasc Disord.

[37] Jafri SH, Imran TF, Medbury E, Ursillo J, Ahmad K, Imran H, Drwal K, Wu W (2022). Cardiovascular outcomes of patients referred to home based cardiac rehabilitation. Heart Lung.

